# Characteristics and occupational exposure assessment of VOCs in different coal mines

**DOI:** 10.3389/fpubh.2025.1705543

**Published:** 2025-12-10

**Authors:** Xiaozhou Liu, Sheng Xue, Bingjun Liu, Jingbei Zhang, Yuchen Xie

**Affiliations:** 1 School of Earth and Environment, Anhui University of Science and Technology, Huainan, Anhui, China; 2 Joint National-Local Engineering Research Centre for Safe and Precise Coal Mining, Anhui University of Science and Technology, Huainan, Anhui, China; 3 Huainan Academy of Atmospheric Sciences, Huainan, Anhui, China

**Keywords:** volatile organic compounds (VOCs), descent method, VOCs concentration, VOCs characteristics, occupational exposure assessment

## Abstract

**Introduction:**

The occupational health risk to miners from exposure to volatile organic compounds (VOCs) in underground coal mines has not been adequately quantified. This study aimed to investigate the pollution characteristics and assess the health risks of VOCs in three typical mining areas.

**Methods:**

VOC concentrations and compositions were monitored and analyzed across different locations (working face, air return tunnel, air intake tunnel) in three distinct mines: Pan San Mine, Qinghai Yuqa Mine, and Hongliulin Mine, which employed different mining and transport methods.

**Results:**

The main findings were: ① Underground VOC concentrations substantially exceeded surface levels, with peaks of 10.8 mg/m^3^ (Pan San), 9.31 mg/m^3^ (Qinghai Yuqa), and 14.29 mg/m^3^ (Hongliulin) at the working face. A consistent spatial pattern (working face > air return tunnel > air intake tunnel) identified the face as the primary source. ② VOC composition varied with mining methods. In mines using cage elevators or monkey cars, alkane proportions increased by 18.7–23.4% with depth, while OVOCs decreased by 12.1–15.6%, indicating dominant natural emissions from coal seams. In contrast, the mine using diesel transport showed minimal variation (≤4.8%), highlighting the strong influence of mining activities.

**Discussion/Conclusion:**

The analysis revealed that for the limited subset of VOCs with established occupational exposure limit values (17 of 116), exposure indices were significantly below 1. Furthermore, exposure to the remaining compounds was also negligible. These results indicate that, under the studied conditions, the observed VOC levels did not pose a significant occupational health risk.

## 1 Introduction

Air quality in underground coal mines has long been a critical concern in occupational safety and health research (1–5). The confined nature of these environments facilitates the accumulation of hazardous substances such as coal dust and methane, posing direct threats to miners’ health. While traditional studies have focused on conventional pollutants (6–10), research on health risks associated with volatile organic compounds (VOCs) remains in its nascent stage.

Volatile organic compounds (VOCs)—a group of organic compounds that include alkanes, alkenes, and aromatic hydrocarbons—originate from industrial emissions, fuel volatilization, and other anthropogenic sources. Current research exhibits a bimodal focus: macroscopic investigations address VOC composition, source apportionment, and ozone/secondary organic aerosol (SOA) formation in open atmospheres (11–19). Hu et al. (20) identified plant emissions (41.1%), industrial processes (32.6%), and solvent use (14.3%) as dominant VOC sources during pollution episodes through PMF modeling, with particular monitoring priorities for isoprene and aromatic hydrocarbons. Contrastingly, Yan et al. (21) demonstrated distinct source patterns in Dalian: industrial areas showed higher contributions from fossil fuel evaporation (32.8%) and solvent use (31.4%), while residential areas were predominantly influenced by vehicle emissions (40.2%). Notably, solvent use accounted for 57.4% of ozone formation potential (OFP) in industrial zones, versus 40.8% from vehicle sources in residential areas, highlighting the necessity for differentiated control strategies across functional zones.

Recent investigations have increasingly prioritized semi-confined spaces such as traffic tunnels and underground parking facilities (11, 18, 22–28). Occupational exposure to hazardous substances presents cross-industry health risks, as evidenced by diesel exhaust-associated cardiovascular risks and silica/metals exposure in manufacturing (29–32). Similarly, coal miners face complex VOC exposures originating from both natural sources (e.g., geogenic emissions from coal seams) and anthropogenic activities (particularly diesel-powered equipment operations).

Notably, research on VOCs in coal mine subsurface environments remains conspicuously underdeveloped. Distinct from conventional confined spaces, mine atmospheres feature dual emission mechanisms—natural release (coalbed desorption) and anthropogenic sources (mechanical operations)—coupled with complex ventilation dynamics. This study addresses this gap through a tri-regional investigation of underground coal mines. Employing Teflon sampling bags for ambient air collection under diverse operational conditions, we analyzed VOC concentration profiles and compositional variations via gas chromatography–mass spectrometry (GC–MS). The EU occupational exposure assessment methodology, which uses Occupational Exposure Limits and exposure index calculations, was applied to conduct a quantitative evaluation of chemical risks in three Chinese mines. Our findings provide critical baseline data and theoretical foundations for improving underground air quality. Future research should elucidate the mechanistic impacts of environmental factors (e.g., temperature, humidity, ventilation efficiency) on VOC migration and transformation processes.

## 2 Sampling and analysis methods

### 2.1 Sampling locations and processes

This study conducted systematic volatile organic compound (VOCs) sampling campaigns across three representative mining areas (Hongliulin Coal Mine, Pan San Mining Company, and Qinghai Yuka Mine). All sites implemented standardized sampling protocols: initial ground-level background monitoring stations were established at mine entrances to obtain baseline concentration data, followed by spatial gradient sampling along predefined pathways (connecting tunnel → air intake tunnel → working face → air return tunnel) using distinct transportation methods (diesel-powered vehicles via inclined shafts at Hongliulin, cage hoists through vertical shafts at Pan San, and monorail suspended vehicles in inclined shafts at Qinghai). Teflon sampling bags were employed for fixed-point collection in typical operational microenvironments, yielding 59 valid samples (Hongliulin 19, Pan San 20, Qinghai 20) with strict adherence to quality control protocols. This stratified sampling strategy comprehensively covered three-dimensional mine ventilation networks, establishing a robust dataset for investigating VOCs migration patterns within excavation systems.

**Figure 1:**
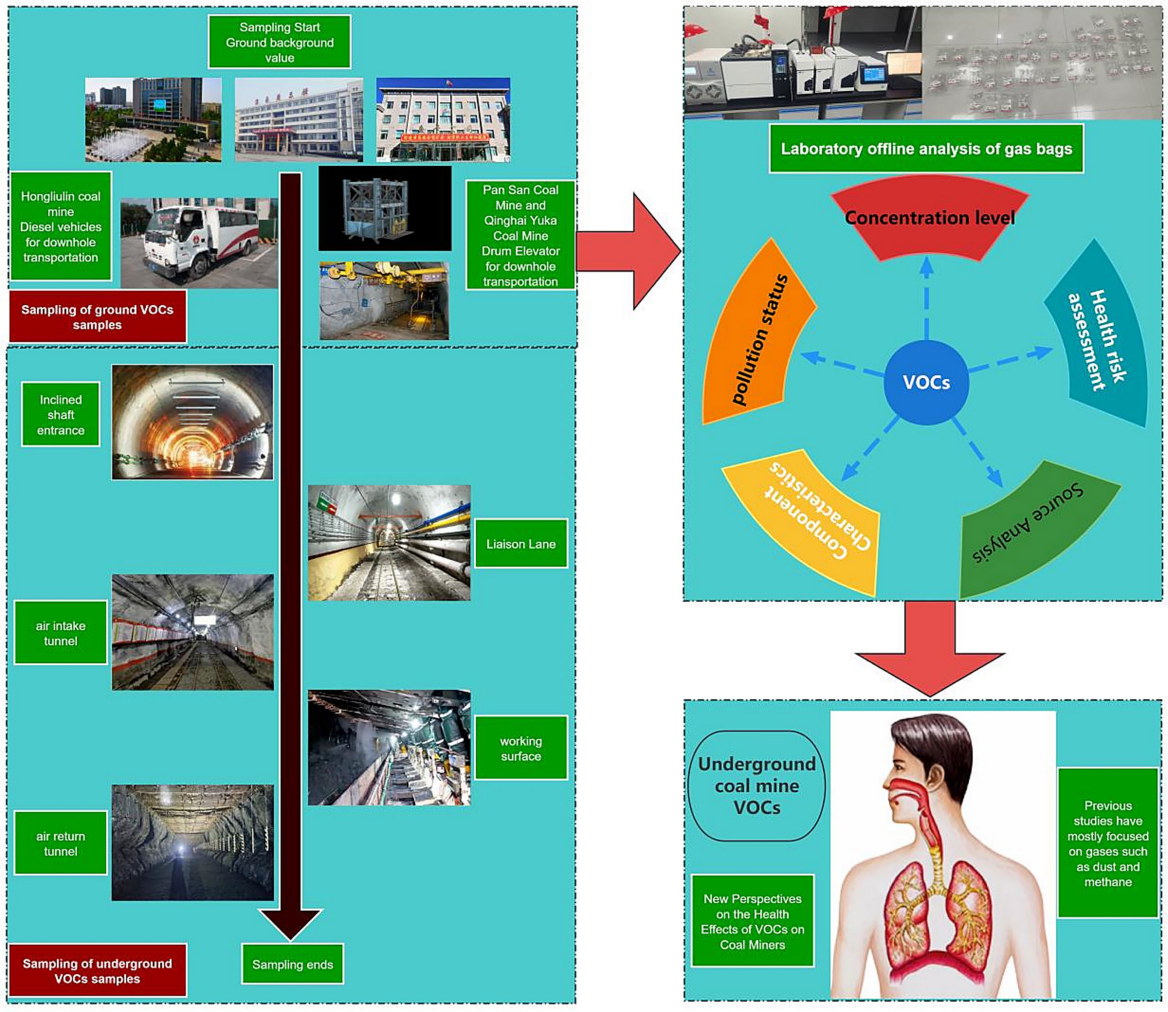
Sampling process schematic diagram.

At Hongliulin Coal Mine, the VOCs sampling initiative specifically involved establishing monitoring points at the mine entrance to capture surface background values as reference data. Diesel-powered transport vehicles were utilized to access underground workings through inclined shafts, progressively sampling along connecting tunnels, intake airways, working faces, and return airways. Nineteen representative VOCs samples were meticulously collected across these distinct operational zones to ensure comprehensive spatial coverage and analytical reliability. Table 1 shows the transportation modes for going down the well and Figure 1 shows the sampling schematic diagram.

**Table 1:** Downhole transportation.

Name of coal mine	Coal mine entrance	Means of transportation
Hongliulin	Inclined shaft	Diesel vehicle
Pan San	Vertical shaft	Cage filling elevator
Qinghai Yuka	Inclined shaft	Coal mine monkey cart

### 2.2 Sample collection and analysis

Due to safety regulations prohibiting the use of active personal sampling pumps underground, this study employed area sampling as a pragmatic alternative to assess volatile organic compounds (VOCs). Sampling was conducted using 1-liter Teflon bags at fixed locations in critical functional zones (e.g., working face, return airway), with inlets positioned at approximately 1.5 m above ground to approximate the human breathing zone.

While general quality control procedures for bag sampling—such as those outlined in the stationary source standard HJ 732-2014—were adopted, this method was explicitly adapted for atmospheric characterization in mines and was not intended to quantify personal exposure doses. Samples were protected from light after collection and analyzed within 72 h to maintain stability. The resulting area concentration data serve as a conservative estimate for preliminary screening of exposure levels and should be interpreted as semi-quantitative in the context of this exploratory study.

The VOC samples were analyzed on a Pano AMD 10 GC–MS analyzer. After enrichment and dissolution, the samples were separated on a chromatographic column and then analyzed by mass spectrometry (MS) for qualitative and quantitative analysis. Pre-treatment conditions: the sample flow rate of the adsorption tube was 20 mL/min, the sample volume was 100 mL, the inlet temperature was 120 °C, the thermostat temperature was 45 °C, the desorption temperature of the adsorption tube was 300 °C, and the desorption time was 2.0 min. GC conditions: the column used for gas chromatography was DB-1 60 m × 0.250 mm × 1.0 um, Agilent, United States, with damping column: 7 m × 0.250 mm × 0.0 um, and the column was 7 m × 0.250 mm × 1.0 um. Damping column: 7 m × 0.20mm × 0 um was used for the separation of VOCs, and the flow rate of high purity helium as carrier gas was 1.2 mL/min without shunt. The temperature of the forward sample port was 150 °C. The programmed temperature increase was 40 °C for 10 min, then increased to 190 °C at 5 °C/min and held for 8 min. MS conditions: the electron ionization source was selected for mass spectrometry, the ion source temperature was 250 °C, the mass spectrometry transfer line temperature was 250 °C, the gas interface temperature was 230 °C and the mass spectrometry scan mode was full scan with a scan range of 45–300 amu and a scan rate of 2000 amu. FID conditions: the column was Agilent J&W HP-PLOT Al₂O₃KCl 30 m × 0.320 mm × 8.0 um, the FID temperature was 250 °C, the flow rate of hydrogen was 40 mL/min, the flow rate of air was 450 mL/min, and the flow rate of tail blow was 45 mL/min. The sample analytical fractions totaled 116 VOC fractions (Table 2 shows the detection limits and correlation coefficients for the target compound).

**Table 2:** Target compound and correlation coefficients.

Target compound	*R* ^2^	Target compound	*R* ^2^
Dichlorodifluoromethane	0.998530	2-Methylhexane	0.999184
Chloromethane	0.999029	2,3-Dimethylpentane	0.999040
Dichlorotetrafluoroethane	0.998672	Valeraldehyde	0.998063
Isobutane	0.998415	3-Methylhexane	0.999215
Acetaldehyde	0.998901	1,2-Dichloropropane	0.998883
1-Butene	0.998722	Monobromodichloromethane	0.998126
1,3-Butadiene	0.999051	1,4-Dioxane	0.995936
Butane	0.998693	Trichloroethylene	0.995721
2-Butene	0.999076	2,2,4-Trimethylpentane	0.998740
Methyl bromide	0.997814	Methyl methacrylate	0.996877
Cis-2-butene	0.999532	Heptane	0.999010
Ethyl chloride	0.999643	1,3-Dichloropropene	0.997813
Ethane	0.999846	4-Methyl-2-pentanone	0.998102
2-Propenal	0.999096	Methylcyclohexane	0.999154
Acetone	0.998216	trans-1,3-Dichloropropene	0.997250
Propionaldehyde	0.998794	1,1,2-Trichloroethane	0.999136
Isopentane	0.999131	2,3,4-Trimethylpentane	0.998298
Ethylene	0.999742	Toluene	0.988939
Trichloromonofluoromethane	0.999326	Heptane, 2-methyl-	0.999392
Isopropyl alcohol	0.996581	2-Hexanone	0.998629
1-Pentene	0.999338	2,2,3-Trimethylpentane	0.999481
n-Pentane	0.998986	Dibromochloromethane	0.981687
Isoprene	0.999054	Hexanal	0.993006
trans-2-Pentene	0.999234	1,2-Dibromoethane	0.995502
Ethene, 1,1-dichloro-	0.999433	Octane	0.999084
cis-2-pentene	0.999517	Tetrachloroethylene	0.994441
Methylene chloride	0.999483	Chlorobenzene	0.998845
Methane	0.999909	Ethylbenzene	0.999114
1,1,2-Trichloroethane; 1,2,2-Trifluoroethane	0.998510	1,3-Dimethylbenzene	0.999574
Carbon disulfide	0.998099	Tribromomethane	0.993880
2,2-Dimethylbutane	0.999549	Styrene	0.997065
Methacrolein	0.998103	1,1,2,2-Tetrachloroethane	0.998878
trans-1,2-Dichloroethylene	0.998970	o-Xylene	0.999542
1,1-Dichloroethane/ethylidene dichloride	0.999298	Nonane	0.998991
Methyl tert-butyl ether	0.998460	Isopropylbenzene	0.996886
Cyclopentane	0.999488	Benzaldehyde	0.998965
Vinyl acetate	0.999122	n-Propylbenzene	0.995981
2,3-Dimethylbutane	0.998847	m-Ethyltoluene; 1-Ethyl-3-methylbenzene	0.991408
2-Methylpentane	0.998847	p-Ethyltoluene; 1-Ethyl-4-methylbenzene; 4-Ethyltoluene	0.991589
Butanal	0.993816	1,3,5-Trimethylbenzene	0.992433
2-Butanone	0.995008	Benzene, 1-ethyl-2-methyl-	0.992849
3-Methylpentane	0.999469	Benzene, 1,2,4-trimethyl-	0.993851
1-Hexene	0.998767	Decane	0.999656
cis-1,2-Dichloroethylene	0.998782	Benzyl chloride	0.975433
Acrylic	0.999924	Benzene, 1,4-dichloro-	0.991396
Ethyl acetate	0.998307	1,3-Dichlorobenzene	0.992627
n-Hexane	0.999566	1,2,3-Trimethylbenzene	0.989135
Trichloromethane	0.997291	1,2-Dichlorobenzene	0.986082
Tetrahydrofuran	0.998621	m-Diethylbenzene	0.991505
Butenal	0.996191	p-Diethylbenzene	0.995683
1,2-Dichloroethane	0.998242	m-Toluic aldehyde	0.997668
Methylcyclopentane	0.999024	Undecane	0.999381
2,4-Dimethylpentane	0.998999	Benzene, 1,2,4-trichloro-	0.996279
1,1,1-Trichloroethane	0.997582	Naphthalene	0.998871
Benzene	0.995554	Dodecane	0.999632
Propargyl	0.999097	1,3-Butadiene, 1,1,2,3,4,4-hexachloro-	0.997867
Carbon tetrachloride	0.998404	Vinyl chloride	0.999455
Cyclohexane	0.999822		

### 2.3 Risk assessment based on occupational exposure limits

To investigate the health effects of VOCs on workers across different operational scenarios in underground coal mines, this study adopted the European Union’s occupational exposure risk assessment framework (33). The assessment strictly adhered to the EN 689:2018 standard, which employs a systematic measurement strategy to determine whether worker exposure levels are effectively controlled.

Due to safety regulations prohibiting standard electrical equipment in the underground environment—which precludes the use of personal sampling pumps—this exploratory study utilized area monitoring to determine representative VOC concentrations. The measured area concentrations were used as a conservative estimate of the 8-h time-weighted average (TWA) exposure for workers. Following GC–MS analysis, the resulting 8-h TWA exposure concentration for each compound was compared with the corresponding Occupational Exposure Limit Value (OELV) obtained from the IFA database (prioritizing EU values, with US and Chinese standards as secondary references). Risk characterization was performed using the exposure index (EI). An EI < 1 indicates that the exposure level is below the OELV and the risk is considered controlled. Conversely, an EI ≥ 1 signifies that the exposure reaches or exceeds the limit, representing an unacceptable health risk that necessitates immediate implementation of control measures.

### 2.4 Quality control and assurance

Prior to VOCs sampling, rigorous pretreatment procedures were implemented, including purging the gas sampling bags three to four times with high-purity nitrogen (99.999%) to minimize interference from background concentrations. The experiment employed a dynamic dilution apparatus to dilute No. 115 standard gas, generating a 20 ppb standard gas mixture. Different concentration levels of standard gases (2-36 ppb) were obtained by adjusting injection volumes (10-180 mL). Calibration curves demonstrated that standard curve coefficients for target compounds predominantly exceeded 0.99, with a minority reaching 0.98. To ensure data accuracy, each sample underwent dual measurements: initial injection was performed using nitrogen carrier gas to eliminate potential residual contamination, followed by subsequent injection using gas from the sample bag for quantitative determination.

## 3 Results and discussion

### 3.1 VOCs concentration levels

As shown in Table 3, the average concentration levels of total volatile organic compounds (TVOCs) in underground environments of the three mining areas were significantly higher than surface background values (Table 4), exhibiting significant spatial heterogeneity. The mean TVOC concentrations in Hongliulin, Pan San, and Qinghai Yuk mining areas reached 1.5 times (5 vs. 3.39 mg/m^3^), 8.8 times (3.44 vs. 0.39 mg/m^3^), and 7.2 times (4.4 vs. 0.61 mg/m^3^) of their respective surface background values. Notably, a peak concentration of 1.43 mg/m^3^ was recorded at the lower corner of the working face, representing 15.2 times the surface background value and significantly exceeding the Chinese indoor air quality standard threshold (0.600 mg/m^3^). It should be noted that while comparison with indoor air quality standards is not directly applicable to occupational hygiene scenarios, it provides valuable context for understanding the severity of mine air pollution relative to general living environments. For a professional health risk assessment, the core analysis of this study strictly follows the Occupational Exposure Limits framework.

**Table 3:** Air quality standard.

Air quality standard	TVOC (mg/m^3^)
GB/T18883-2002	≤0.6
USA EPA	≤0.5
European Union	≤0.3
Japan	≤0.3

**Table 4:** Ground level background values, TVOC concentrations, and maximum and minimum values.

Name of coal mine	Ground background value	TVOC mg/m^3^	Maximum value of downhole VOCs (mg/m^3^)	Minimum values for downhole VOCs (mg/m^3^)
Hongliulin	3.4	5	1.42	9.25
Pan San	0.39	3.44	1.08	1
Qinghai Yuka	0.61	4.43	9.312	4.18

According to TVOC health risk classification (Table 5), the concentration levels at critical monitoring points (3–14.29 mg/m^3^) have entered the risk range associated with mucosal irritation and neurotoxicity (3–25 mg/m^3^). However, it is important to acknowledge the limitations of the TVOC metric, as it fails to reflect the significant variation in toxicity among individual compounds. Therefore, the TVOC data here serve primarily for initial screening purposes, while the subsequent analysis will focus on detailed assessment of specific compounds, suggesting potential respiratory system disorders under chronic exposure conditions.

**Table 5:** Occupational exposure limits for target volatile organic compounds.

Target compound	OELVs mg·m^−3^	Target compound	OELVs mg·m^−3^
Dichlorodifluoromethane	5,000	Styrene	50
Chloromethane	42	Cyclopentane	1720
Trichloroethylene	54.7	Acrylic	29
Heptane	2,100	Tetrahydrofuran	150
Acetaldehyde	360	1,1,1-Trichloroethane	5
Methylcyclohexane	2000	Benzene	0.66
Toluene	192	Cyclohexane	700
chlorobenzene	23	Naphthalene	50
Ethylbenzene	442		

OELVs were sourced from the GESTIS database.

**Figure 2:**
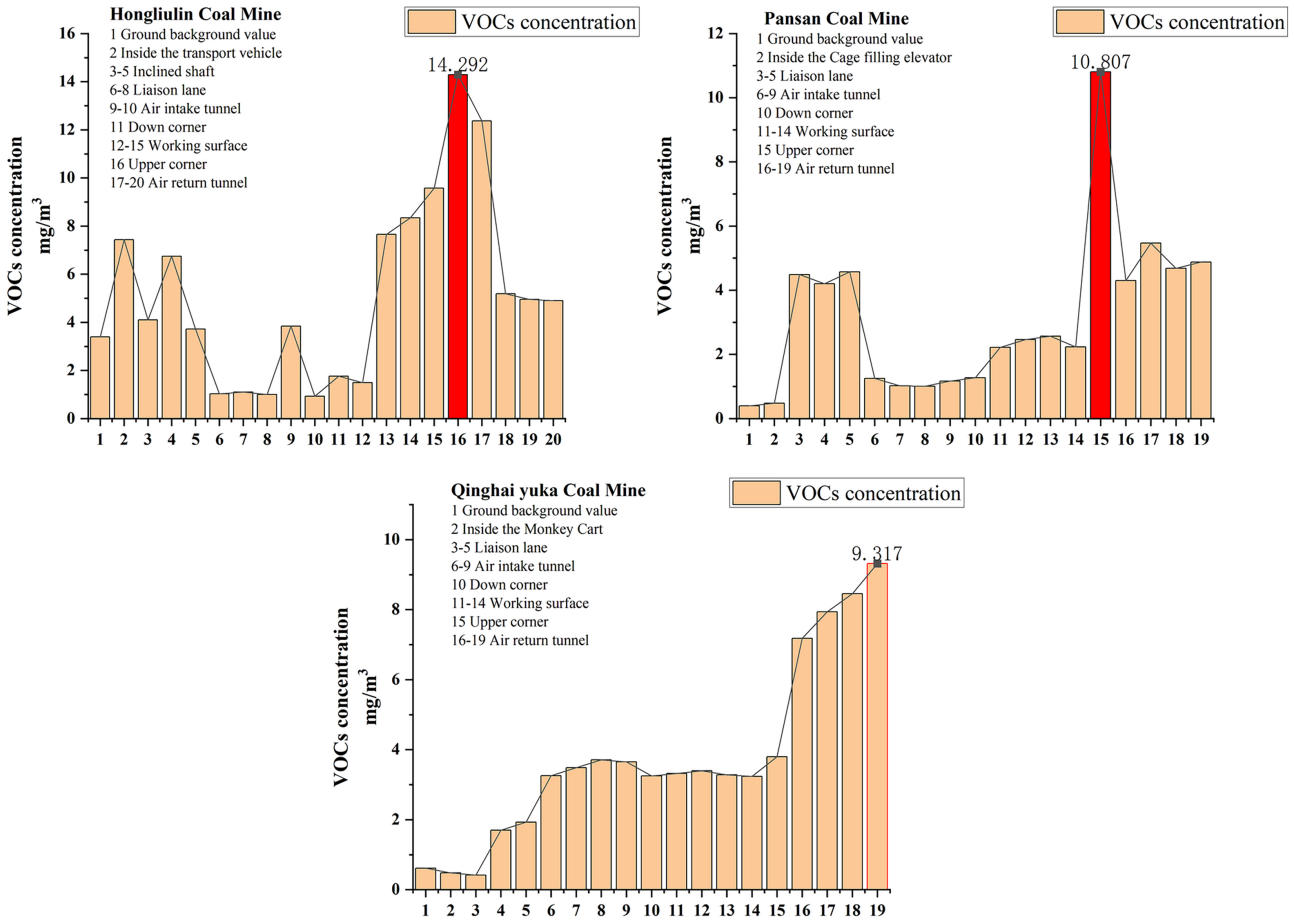
Concentration levels of VOCs in different coal mine.

Figure 2 illustrates a characteristic pollution gradient in TVOC spatial distribution: working face > air return tunnel > air intake tunnel. The working face, functioning as the primary pollution source area, demonstrated localized high-concentration accumulation (Hongliulin: 14.29 mg/m^3^) due to combined effects of coal-cutting operations (shearing and transportation) and coal matrix desorption. The air intake tunnel maintained relatively low TVOC concentrations (0.92–1 mg/m^3^) through continuous fresh air supply from high-efficiency ventilation systems. Serving as pollution dispersion channels, air return tunnel exhibited decreasing TVOC concentrations along airflow pathways (9.31 → 3.45 mg/m^3^), demonstrating the synergistic dilution-emission effect of ventilation systems. This distribution pattern reveals that the source-sink relationship of underground VOCs is jointly regulated by mechanical disturbance intensity and ventilation efficiency.

The findings indicate that prolonged occupational exposure to high-concentration VOCs at working faces poses significant health risks to miners. We recommend: ① establishing a dynamic VOCs monitoring system specifically for coal mines, with prioritized surveillance at working faces and return airways; ② developing differentiated VOCs concentration limits based on mechanical operation intensity and ventilation parameters; ③ prioritizing toxicological research on high-risk components like benzene homologues and aldehyde-ketone compounds. This study provides fundamental data for constructing VOCs migration models in underground multiphase media and formulating engineering control measures.

### 3.2 Composition and characterization of VOCs

In the study of volatile organic compounds (VOCs), it has been found that VOCs can be further subdivided into a number of classes based on their molecular structure and reactivity with hydroxyl radicals (OH radicals). These categories include alkynes, alkanes, alkenes, aromatic hydrocarbons, oxygenated volatile organic compounds (OVOCs), and halogenated hydrocarbons. Each of these different categories has a unique chemical structure and properties. As shown in Figure 2, by classifying the results obtained from the component analysis of the collected samples, we can observe that the trend of the percentage of the categories in Pan San Mine and Qinghai Yuqa Coal Mine shows some consistency. In particular, as the sampling process progresses, the percentage of alkanes gradually increases, while the percentage of oxygenated volatile organic compounds (OVOCs) gradually decreases. This trend can be attributed to the fact that the main source of VOC emissions from these two coal mines are VOCs generated by volatilization from the coal seam itself, while the proportion of OVOCs, as secondary metabolites, gradually decreases due to the lack of more anthropogenic emission sources. In addition, the proportions of aromatic hydrocarbons, olefins and alkynes also gradually decreased with the sampling process, which is related to the increasing depth of underground mining in coal mines, and the contribution of anthropogenic sources to VOCs became smaller and smaller. However, the trend of the percentage of VOCs from Hongliulin Coal Mine was completely different. This may be due to the fact that among the three mining methods, only Hongliulin Coal Mine uses diesel trucks to directly enter the inclined shaft and transport miners to the connecting road and the working face. The diesel vehicle exhaust is emitted as artificial source VOC emissions, which is transported by the ventilation system of the entrance road and circulated to the working face and return road until it is discharged from the air shaft. As for the two coal mines, Pan San Mine and Qinghai Yuqa Mine, one of them used the irrigation lift to reach the haul road directly and the other one used the monkey car to reach the haul road without any more artificial source emissions, thus showing a trend of increasing the proportion of alkanes and gradually decreasing the proportion of oxygenated volatile organic compounds (OVOCs) and other types. The Red Willow Grove mine shows little change in the overall percentage trend due to the contribution of diesel exhaust emissions. In our previous studies of VOCs in confined spaces, we found that aromatic hydrocarbons were the largest contributors to VOCs among the components of VOC pollution in tunnels, subways and underground car parks. This is mainly due to the fact that the main means of transport in tunnels and underground car parks are motor vehicles, which have the highest proportion of aromatic hydrocarbons in the pollutant gases emitted from their exhaust. Coal mines, on the other hand, do not normally use petrol vehicles for underground operations, so there are no petrol vehicle exhaust emissions, which also confirms the conclusion that the contribution of aromatic hydrocarbons from the three coal mines is low in the analysis results of this study.

In summary, significantly differing from closed-space environments like tunnels and subways (where aromatic hydrocarbons account for >40%), coal mines in this study showed an average aromatic content of merely 6.8 ± 2.3%. This discrepancy stems from specialized operational protocols: the prohibition of gasoline vehicles underground effectively eliminates the primary input pathway of benzene-series compounds from exhaust emissions, confirming the strong correlation between anthropogenic contribution rates (Hongliulin: 34.7% vs. other mines: <8.2%) and transport vehicle types in source apportionment.

### 3.3 Risk assessment based on occupational exposure limits value

To quantitatively evaluate the potential health risks to miners, an occupational exposure assessment was conducted for the detected VOCs following the European Union methodology. Out of the 116 compounds identified through GC–MS screening, 17 substances had established Occupational Exposure Limit values available in the GESTIS international database (33) (see Table 5). The risk was characterized using the exposure index (EI), calculated as the ratio of the measured exposure concentration (E) to the corresponding OELV (EI = E/OELV). An EI value of less than 1 indicates that the exposure level is below the regulatory threshold of concern.

**Figure 3:**
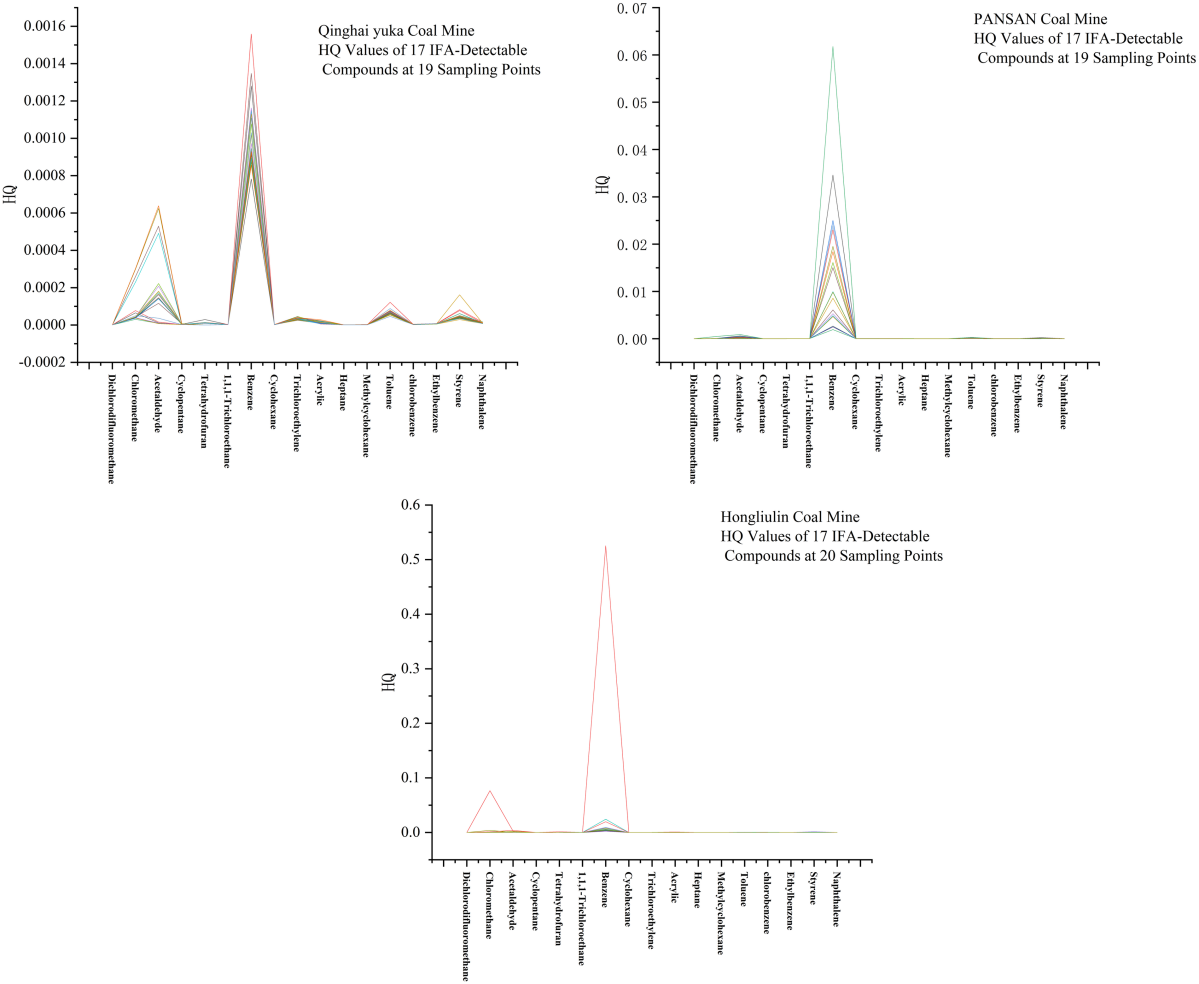
Classification and composition of VOCs in different work environments of coal mines.

**Figure 4:**
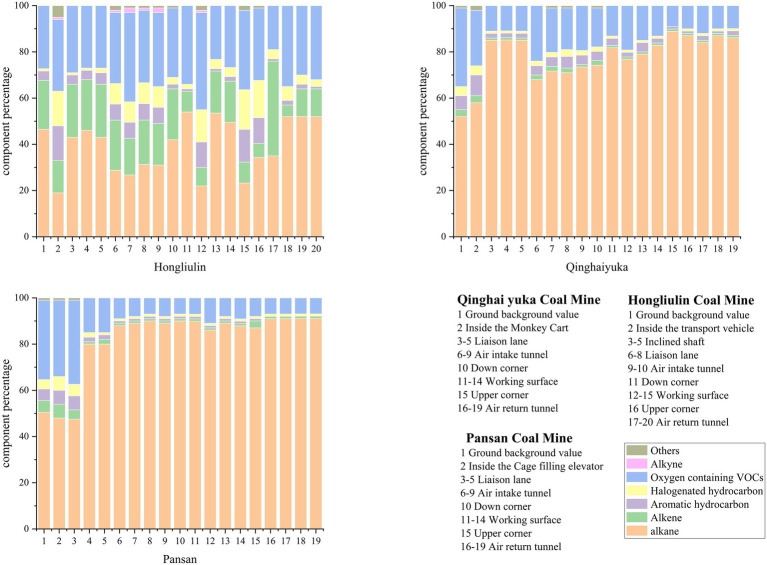
Classification and composition of VOCs in different coal mines.

The calculated EI values for these 17 compounds across all monitoring points in the three mines are summarized in Figure 3. Critically, all EI values were significantly below 1, with the vast majority falling orders of magnitude lower. This demonstrates that, for the limited set of compounds with defined regulatory limits, the measured concentrations do not pose a significant occupational health risk. However, a crucial finding of this study lies in the composition profile detailed in Figure 4. The VOC mixture was overwhelmingly dominated by light alkanes and alkenes (e.g., propane, ethane, ethylene), primarily generated through natural geogenic processes like coal seam desorption. This absence likely reflects that these substances have not been a primary occupational health concern in conventional industrial settings, or that sufficient occupational health data for standard-setting is lacking.

Consequently, a standard risk assessment cannot be performed for the very compounds that constitute the bulk of the miners’ long-term VOC exposure background in this unique, confined environment. This limitation underscores the exploratory value and necessity of our study. The primary objective is not merely to monitor compounds with known risks, but to first clarify the complete spectral composition of VOCs in coal mines, especially these “underexplored” substances. Our systematic screening and classification work provides the essential foundational dataset required to identify priority components for future toxicological studies and the potential development of relevant exposure limits.

A finding that validates the sensitivity of our approach was observed in the Hongliulin Mine. As the only mine employing diesel-powered transport vehicles underground, it showed discernibly elevated—though still sub-threshold—EI for benzene, toluene, and acetaldehyde compared to the other mines. This pattern confirms diesel exhaust as an anthropogenic source and reinforces the reliability of our data, highlighting that machinery use is a key factor altering the VOC profile.

In conclusion, while the risk from compounds with existing OELV is low, the inability to assess the majority of the VOC background emphasizes the need for continued research. This study lays the indispensable groundwork for future targeted occupational health programs in this specific environment.

## 4 Conclusion

The findings of this study demonstrate:

(1) Underground VOC concentrations were significantly higher than surface background levels, exhibiting a spatial gradient: working face > air return tunnel > air intake tunnel. As the primary pollution source area, the working face showed average VOC concentrations 4.2–15.2 times higher than surface background values, indicating severe VOC accumulation in this zone. (2) Distinct geochemical differentiation of VOC components was observed between Pan San Mine and Qinghai Yukai Mine. With increasing sampling depth, alkane proportions increased (+18.7–23.4%) while oxygenated VOCs (OVOCs) decreased (−12.1–15.6%), suggesting dominant volatilization of primary coalbed VOCs. In contrast, Hongliulin Mine exhibited ≤4.8% fluctuations in alkane and OVOC proportions due to diesel vehicle transportation, confirming substantial anthropogenic impacts on VOC composition. (3) The risk assessment for compounds with established OELVs (17 out of 116 detected) indicated no significant occupational exposure (EI << 1). For the majority of the VOC profile, analysis of the samples from this exploratory study determined that the measured exposures were negligible and did not warrant further follow-up investigation.

## Data Availability

The original contributions presented in the study are included in the article/supplementary material, further inquiries can be directed to the corresponding authors.
